# The relationship between demographic- and disease-related variables and health-related quality of life in patients with axial spondyloarthritis

**DOI:** 10.1186/s12891-017-1693-z

**Published:** 2017-08-01

**Authors:** Gudrun Rohde, Kari Hansen Berg, Anne Prøven, Glenn Haugeberg

**Affiliations:** 1 Faculty of Health and Sport Sciences, University of Agder, Kristiansand, 422, 4604 Kristiansand, Norway; 2 Department of Clinical Research, Sorlandet Hospital, Kristiansand, 4604 Norge; 3 Marie Curie Palliative Care Research Department and Division of Psychiatry, University College London, London, UK; 4 Department of Rheumatology, Martina Hansens Hospital, Bærum, Norway; 5 Department of Neurosciences, Rheumatology Division, INM, Norwegian University of Science and Technology, Trondheim, Norway

**Keywords:** Axial spondyloarthritis, Health-related quality of life, Utility measures, Exercise, Targeted medications

## Abstract

**Background:**

Axial spondyloarthritis (ax-SpA) is a chronic inflammatory disease of the spine causing pain, stiffness, loss in physical function, and fatigue. Therefore, the physical and psychological burden of having this chronic disease can reduce the quality of life. We aimed to explore the relationship between demographic- and disease-related variables and health-related quality of life (HRQoL) in patients with ax-SpA.

**Methods:**

Demographic- and disease-related, HRQoL-related and treatment data were collected. Disease measures included the Bath Ankylosing Spondylitis Disease Activity Index (BASDAI), the BAS Functional Index (BASFI), the BAS Global (BAS-G) score, the Maastricht Ankylosing Spondylitis Enthesitis Score (MASES), the Health Assessment Questionnaire (HAQ) and co-morbidity. HRQoL was assessed using the SF-36 and the utility measures SF-6D and 15D. Variables associated with HRQoL were identified in unadjusted and adjusted analyses.

**Results:**

We examined 380 patients with ax-SpA (67% men) with a mean age of 46 years. Among them, 86% reported exercising >1 h per week. Mean values were as follows: BASDAI, 3.17; MASES, 3.19; BASFI, 2.71; BAS-G. 3.88; and HAQ, 0.56. The percentage of current users of NSAIDs was 44%, and of DMARDs 23%. In multivariate analyses, exercising 1–3 h per week (B = 2.73, *p* = 0.022) and exercising >3 h per week (B = 2.71, *p* = 0.020), lower HAQ scores (B = −4.61, *p* = 0.001), lower BASFI scores (B = −1.05, *p* = 0.010) and lower BAS-G scores (B = −0.91, *p* = 0.001) were independently associated with higher SF-36-PCS scores, whereas modest alcohol consumption (B = 4.63, *p* = 0.018) and a lower BAS-G score (B = −1.73, *p* < 0.001) were independently associated with higher SF-36-MCS scores. Exercising 1–3 h per week (B = 0.032, *p* = 0.004) and exercising >3 h per week (B = 0.036, *p* = 0.001), lower HAQ scores (B = −0.051, *p* < 0.001), lower BAS-G scores (B = −0.010, *p* < 0.001) and co-morbidity (B = −0.014, *p* = 0.004) were independently associated with higher 15D scores. Finally, exercising 1–3 h per week (B = 0.045, *p* = 0.001) and exercising > 3 h per week (B = 0.053, *p* < 0.001), lower HAQ scores (B = −0.054, *p* = 0.001) and lower BAS-G scores (B = −0.020, *p* < 0.001) were associated with higher SF-6D scores.

**Conclusions:**

In patients with ax-SpA, a low level of physical activity, impaired physical function and impaired general well-being were independently and consistently associated with a decreased HRQoL across all applied measures.

## Background

Axial spondyloarthritis (Ax-SpA) is a chronic, systemic inflammatory rheumatic disease affecting the axial skeleton. Its inflammatory disease processes can also involve peripheral joints, entheses and internal organs [1, 2]. Most frequently, the disorder presents with an insidious onset of back pain in early adulthood, and typically causes fatigue, stiffness and loss in physical function [2, 3]. The psychological burden of having such a chronic disease in addition to the somatic symptoms can have a major impact on the health-related quality of life (HRQoL) in patients with ax-SpA, both for patients with ankylosing spondylitis (AS) and non-radiographically determined ax-SpA, even at a young age [4–7]. HRQoL is a subjective and multidimensional concept and can be defined as an individual’s experience of their general health state, such as physical, social and mental well-being [8].

Data from the literature indicate no differences in HRQoL between patients with AS and non-radiographically determined ax-SpA [9]. Furthermore, patients with ax-SpA have reported HRQoL scores that are similar to other inflammatory diseases such as psoriasis and inflammatory bowel disease [10, 11]. However, patients with ax-SpA report lower HRQoL than do healthy controls [3, 12], and women with the disease report a lower HRQoL than do men [2, 13, 14]. Decreased HRQoL in patients with ax-SpA is associated with fatigue [15], increased disease activity, decreased daily activity and exercise [16–18], pain, and adverse psychological factors such as body image disturbance, anxiety and depression [6, 19].

HRQoL measurements can be used for economic evaluation (cost–utility analysis), and several generic utility instruments (e.g., 15D, SF-6D and EQ5D) [20, 21] have been developed. The utility measures can also be used to calculate quality-adjusted life years (QALYs) [20, 21]. Furthermore, HRQoL can be assessed using other generic HRQoL measures such as the well-known SF-36 [22, 23].

There is sparse knowledge on the associations between demographic- and disease-related variables and HRQoL using utility measures in patients with ax-SpA, especially after the introduction of new and more targeted medications [24]. Therefore, the aim of this study was to explore the relationships between demographic- and disease-related variables and HRQoL in patients with ax-SpA, using the utility measures 15D and SF-6D and the generic HRQoL measure SF-36.

## Methods

### Patient recruitment

Patients with ax-SpA included in this study were recruited consecutively from two outpatient rheumatology clinics, one located in the eastern part of Norway (Martina Hansens Hospital, MHH) and the other in the southern part (Sorlandet Hospital, SSHF), which also have been described previously [25]. The patients had to be 18 years or older and needed to meet the Assessment of Spondyloarthritis International Society (ASAS) criteria for ax-SpA [26].

### Data collection

A broad spectrum of demographic, disease- and treatment-related data was collected using physical examinations, laboratory tests, interviews and questionnaires [25]. Demographic data included age, gender, education level, work status, physical exercise, body mass index (BMI), smoking and alcohol consumption. Disease duration was defined as the time between the date fulfilling the ASAS criteria for ax-SpA and the date for inclusion in the study, and human leucocyte antigen (HLA)-B27 status was registered. Disease activity was assessed using the Bath Ankylosing Spondylitis Activity Index (BASDAI), the Maastricht Ankylosing Spondylitis Enthesitis Score (MASES) and C-reactive protein (CRP) level. Physical function was assessed by the Bath Ankylosing Spondylitis Functional Index (BASFI) and the Health Assessment Questionnaire (HAQ) [27]. Data on the Bath Ankylosing Spondylitis Patient Global Score (BAS-G) and morning stiffness were also collected. Any additional current medications including the use of non-steroidal anti-inflammatory drugs **(**NSAIDs), synthetic disease-modifying anti-rheumatic drugs **(**DMARDs), biological DMARDs and prednisolone were also recorded. Data on co-morbidities (yes/no) (heart diseases, pulmonary diseases; neurological, endocrine, haematological, gastro-intestinal, urogenital, other rheumatology diseases, mental disorders and cancers) were collected, and we computed a summed score to reflect co-morbidity. This score has also been used in other studies [25, 28, 29].

HRQoL was assessed using both the SF-36 and 15D tools, which also has been described previously [30]. The former is a self-reported and generic questionnaire, including eight domains: general health, bodily pain, physical function, role limitations (physical), mental health, vitality, social function and role limitations (emotional). The eight domains can be combined into a physical and mental sum scale that reflects physical and mental health. The physical component summary (PCS) and the mental component summary (MCS) scales were also used in this study. Regression analyses were performed to impute missing values in accordance with the instruction by the developer of the questionnaire [22, 23]. The SF-36 scales were scored according to published scoring procedures, and each scale was expressed using values from 0 to 100, with 100 representing excellent health [22, 23, 31, 32].

From the SF-36 score we also generated the utility measure SF-6D [21]. This is based on 11 questions from the SF-36 and includes six dimensions, each with four to six levels. The SF-6D utility scores range from 0.29 to 1.00, with 1.00 indicating “full health”. The Norwegian standard SF-36 version 1.00 was used to derive the SF-6D. Regression analyses were performed to impute missing values in accordance with instructions published by the developer of the questionnaire [21, 22]. The SF-6D questionnaire has been validated for its psychometric properties in other studies in several countries [21].

The 15D questionnaire is a generic, multidimensional, standardized evaluation tool of HRQoL that can be used primarily as a single index measure, but also as a profile utility measure, which has been described previously [28]. It describes the patient’s health status, assessing 15 dimensions: mobility, vision, hearing, breathing, sleeping, eating, speech, elimination, usual activities, mental function, discomfort and symptoms, depression, distress, vitality and sexual activity [20]. Each dimension comprises one question with five response categories. A single utility index score is obtained by incorporating population-based preference weights to the dimensions [33, 34]. The utility scores fall between 0.0 (being dead) and 1.00 (no problems on any dimension). Regression analyses were performed to impute missing values in accordance with the guidelines published by the developer of the questionnaire [20]. The questionnaire has been validated thoroughly for psychometric properties in other studies in several countries [20, 33, 34].

### Statistical analyses

Statistical analyses were performed using IBM SPSS Statistics (IBM Corp., Armonk, NY, USA (version 22)). Continuous variables are presented as the mean and standard deviation (SD, in parentheses) and categorical variables as numbers and proportions (%). Chi-squared tests and Student’s *t* tests were used to compare differences between subgroups. To further examine the differences in SF-36 scores between our patients and norm-based scores, we calculated the effect size, calculated by subtracting the mean SF-36 scores in the patients from the mean SF-36 scores in the general population and then dividing by the SD for the general population [35]. We applied Cohen’s standards for effect size values as: small effect 0.2; medium effect 0.5; and large effect 0.8 [36].

Multiple linear regression analysis (general linear model (GLM) in SPSS) was used to examine the adjusted association between demographic- and disease-related variables and HRQoL (SF-36-PCS, SF-36-MCS, 15D and SF-6D scores). The independent variables in the multiple analyses were chosen based on univariate associations with HRQoL, clinical experience and factors associated with HRQoL in previous studies [6, 15, 37]. In the final multivariate model, we included the demographic variables, disease activity (assessed by BASDAI and MASES scores), health status (assessed by HAQ, BASFI and BAS-G scores), damage (assessed by BASMI score), co-morbidity and treatment centre as independent variables. Treatment, disease duration and morning stiffness were also tested in the models as independent variables; however, the same patterns persisted when exploring these associations with HRQoL as dependent variables, so were excluded. The final tested variables are listed in Table 3. For robustness, we also tested the models by backward multiple regression analyses. The level of significance was set at *p* < 0.05.

### Ethical and legal aspects

The study was approved by the Regional Committee for Medical Research Ethics (REK # 4.2007.2152).

## Results

### Demographic- and disease-related characteristics

In total, 389 patients with ax-SpA were included in the study. Among these, 380 gave valid responses to the main HRQoL measure SF-36, and comprised the final sample. The only significant difference between the non-responders (*n* = 9) and responders to SF-36 was age (57.7 (SD 7.1) vs. 45.5 (12.0) years (*p* = 0.001).

When comparing demographic variables listed in Table 1 between the two centres, MHH (*n* = 252) and SSHF (*n* = 128), a statistically significant difference was identified for duration of education >13 years (60% vs. 49%; *p* = 0.038). Concerning disease-related variables, patients from MHH had better outcomes compared with SSHF in terms of BASDAI (2.9 (2.1) vs. 3.7 (2.1), *p* = 0.001); BASFI (2.5 (2.2) vs. 3.0 (2.3), *p* = 0.045); BAS-G (3.6 (2.5) vs. 4.4 (2.6), *p* = 0.009); and MASES (2.4 (2.90) vs. 4.90 (4.66), *p* < 0.001). Patients from MHH currently used more biological DMARD treatment than those from SSHF (30% vs. 9%, *p* < 0.001). For the remaining variables in Table 1, there were no significant differences between the centres. For the analysis, we pooled the patient data and adjusted for the two centres in multivariate analyses.

**Table 1:** Demographic factors, co-morbidity, disease markers, disease activity measures, damage and health status in an axial spondyloarthritis outpatient clinic

	Total (*n* = 380)			
	All	Women (*n* = 127) 33%	Men (*n* = 253) 67%	p
Demographic factors				
Age (years)	45.8 (12.0)	45.8 (12.4)	45.4 (11.8)	0.754
Married/cohabiting	286 (75%)	94 (74%)	192 (76%)	0.642
Employed	263 (73%)	81 (67%)	185 (74%)	0.088
Education				0.736
≤ 10 years	42 (12%)	13 (10%)	30 (12%)	
11–13 years	122 (32%)	39 (31%)	83 (33%)	
> 13 years	213 (56%)	75 (59%)	138 (55%)	
Exercise <1 h per week	54 (14%)	16 (13%)	38 (15%)	0.523
Exercise 1–3 h per week	132 (35%)	47 (37%)	85 (34%)	
Exercise >3 h per week	191 (51%)	63 (50%)	128 (51%)	
Current smoker	105 (28%)	36 (28%)	69 (28%)	0.861
Alcohol consumption (per week)				**0.023**
Never	73 (19%)	33 (26%)	40 (16%)	
1–6 glasses	263 (70%)	84 (67%)	179 (71%)	
7 glasses or more	41 (11%)	9 (7%)	33 (13%)	
BMI (kg/m^2^)	26.9 (4.6)	25.5 (4.7)	27.5 (4.5)	**<0.001**
Disease duration (years)	13.8 (11.4)	12.6 (11.2)	14.4 (11.5)	0.158
Disease marker				
HLA-B27 positive	331 (90%)	107 (87%)	224 (91%)	0.182
Disease activity measures				
CRP (mg/dl)	8.58 (11.94)	7.89 (13.25)	8.2 (11.24)	0.446
BASDAI (0–10)	3.17 (2.11)	3.46 (2.06)	3.03 (2.12)	0.059
MASES (0–13)	3.19 (3.75)	4.41 (3.85)	2.57 (3.54)	**<0.001**
Health status				
Morning stiffness				0.663
< 30 min	230 (60%)	76 (62%)	148 (59%)	
> 31 min	151(40%)	47 (38%)	101 (41%)	
BASFI (0–10)	2.71 (2.23)	2.77 (2.24)	2.68 (2.22)	0.740
BAS-G (0–10)	3.88 (2.57)	4.03 (2.51)	3.81 (2.61)	0.447
HAQ (0–3)	0.56 (0.49)	0.64 (0.53)	0.52 (0.47)	0.034
Damage score				
BASMI (0–10)	2.39 (2.41)	2.03 (1.60)	2.38 (2.19)	**0.013**
Current treatment				
NSAIDs	167 (44%)	59 (47%)	108 (43%)	0.485
Synthetic DMARDs	19 (5%)	8 (6%)	11 (4%)	0.410
Biological DMARDs	88 (23%)	23 (18%)	65 (25%)	0.098
Prednisolone	17 (4%)	7 (5%)	10 (4%)	0.535
Co-morbidity				
Mean total score for co-morbidity (range 0–10)	0.7 (0.9)	0.7 (0.9)	0.6 (0.9)	0.542

Continuous variables are presented as the mean and standard deviation (SD) and categorical variables as number and percentage (%). Bold *p* values indicate significant differences

Key: *BMI* body mass index, *CRP* C-reactive protein, *BASDAI* Bath Ankylosing Spondylitis Disease Activity Index, *MASES* Maastricht Ankylosing Spondylitis Enthesitis Score, *BASFI* Bath Ankylosing Spondylitis Functional Index, *BAS-G* Bath Ankylosing Spondylitis Patient Global Score, *HAQ* Health Assessment Questionnaire, *BASMI* Bath Ankylosing Spondylitis Metric Index, *NSAIDs* non-steroidal anti-inflammatory drugs, *DMARDs* disease-modifying anti-rheumatic drugs

The mean age for the 380 patients was 45.8 (12.0) years; 67% were men; 75% were married or cohabiting; 51% reported exercising >3 h per week and 35% 1–3 h per week. Mean outcome values were as follows: BASDAI 3.17 (2.11); MASES 3.19 (3.75); BASFI 2.71 (2.23); BAS-G 3.88 (2.57); and HAQ 0.56 (0.49). Current users of NSAIDs comprised 44% of the sample versus 23% for biological DMARDs, and 90% were HLA-B27 positive. When comparing women and men with ax-SpA (Table 1), women had a lower BMI (25.5 (4.7) vs. 27.5 (4.5) kg/m^2^; *p* < 0.001) and lower alcohol consumption (1–6 glasses per week (67% vs. 71%), more than 7 glasses per week (7% vs. 13%) *p* = 0.012). Women reported higher MASES (4.42 (3.85) vs. 2.57 (3.53), *p* < 0.001), lower BASMI (2.03 (1.60) vs. 2.38 (2.19), *p* = 0.013) and higher HAQ scores (0.64 (0.53) vs. 0.52 (0.47), *p* = 0.034). For the other variables listed in Table 1, no statistically significant differences were found between men and women.

### HRQoL in patients with ax-SpA

When comparing HRQoL between the MHH and SSHF centres, patients from MHH reported higher scores for the SF-36 domains: vitality (49.5 (20.2) vs. 44.3 (20.0), *p* = 0.019); bodily pain (51.0 (20.0) vs. 43.6 (20.9), *p* = 0.001), physical role limitations (46.7 (41.8) vs. 34.9 (35.5), *p* = 0.014); SF-36-PCS (40.6 (9.1) vs. 37.7 (10.0), *p* = 0.006); and SF-6D (0.67 (0.10) vs. 0.64 (0.10), *p* = 0.003).

When comparing HRQoL between women and men (Table 2), women reported significantly lower scores for the SF-36 domains of bodily pain (45.3 (19.8) vs. 50.1 (20.5), *p* = 0.025) and physical role limitations (36.5 (39.8) vs. 46.5 (41.7), *p* = 0.024). However, when comparing the SF-36 scores in patients with ax-SpA and published norm-based SF-36 reference data for the general population [35], the patients reported significantly lower scores (*p* < 0.001) for all eight SF-36 dimensions. The differences in effect size for the SF-36 dimensions were as follows: mental health −0.20; vitality −0.63; bodily pain −0.90; general health −0.98; social function −0.49; physical function −0.63; physical role −0.89; and emotional role −0.38.

**Table 2:** Health-related quality of life assessed by SF-36, SF-6D and 15D scores in outpatient clinic patients with axial spondyloarthritis, for all subjects, and for women and men separately

	All	Women *n* = 127	Men *n* = 253	p
Health-related quality of life				
SF-36^a^				
Mental health	77.2 (14.6)	77.1 (14.1)	77.2 (14.8)	0.952
Vitality	47.9 (20.3)	44.9 (29.9)	49.2 (19.8)	0.056
Bodily pain	48.5 (20.4)	45.2 (19.8)	50.1 (20.5)	**0.025**
General health	53.8 (21.1)	55.3 (21.3)	53.0 (21.0)	0.336
Social function	75.8 (22.1)	73.9 (22.5)	75.8 (21.8)	0.239
Physical function	73.5 (20.2)	72.4 (19.1)	74.0 (20.8)	0.455
Physical role limitations	43.3 (41.3)	36.5 (39.8)	46.5 (41.7)	**0.024**
Emotional role limitations	72.3 (39.9)	69.7 (42.1)	73.6 (38.7)	0.375
SF-36-PCS	39.6 (9.5)	38.7 (9.2)	40.1 (9.6)	0.169
SF-36-MCS	48.3 (10.2)	47.7 (10.3)	48.5 (10.2)	0.498
SF-6D^b^	0.66 (0.10)	0.66 (0.10)	0.67 (0.08)	0.395
15D^c^	0.85 (0.09)	0.85 (0.08)	0.85 (0.10)	0.859

Data are shown as the mean and (SD)

Key: *SF-36-PCS* physical component summary, *SF-36-MCS* mental component summary. Continuous variables are expressed as the mean and standard deviation (SD). Independent-sample Student’s *t* tests were used for group comparisons. Bold *p* values indicate significant differences

^a^The SF-36 range is 0–100, where 100 indicates a high HRQoL

^b^The SF-6D range is 0–1, where 1 indicates a high HRQoL

^c^The 15D range is 0–1, where 1 indicates a high HRQoL

### Adjusted associations between demographic- and disease-related variables and HRQoL

In the multivariate analyses (Table 3), exercising 1–3 h per week (B = 2.73, *p* = 0.022) and exercising >3 h per week (B = 2.71, *p* = 0.020), lower HAQ scores (B = −4.61, *p* = 0.001), lower BASFI scores (B = −1.05, *p* = 0.010) and lower BAS-G scores (B = −0.91, *p* = 0.001) were independently associated with higher SF-36-PCS values. Modest alcohol consumption (B = 4.63, *p* = 0.019) and lower BAS-G scores (B = −1.73, *p* < 0.001) were independently associated with higher SF-36-MCS. Exercising 1–3 h per week (B = 0.032, *p* = 0.004) and exercising >3 h per week (B = 0.036, *p* = 0.001), lower HAQ scores (B = −0.051, *p* < 0.001), lower BAS-G scores (B = −0.010, *p* < 0.001) and co-morbidity (B = −0.014, *p* = 0.004) were independently associated with a higher 15D score. Finally, exercising 1–3 h per week (B = 0.045, *p* = 0.001) and exercising >3 h per week (B = 0.053, *p* < 0.001), lower HAQ scores (B = −0.054, *p* = 0.001) and lower BAS-G scores (B = −0.020, *p* < 0.001) were associated with higher SF-6D scores. The demographic- and disease-related variables included in the multiple analyses explained 57.1% of the variance in SF-36-PCS, 21.5% in SF-26-MCS, 60.2% in the 15D scores and 56.9% in the SF-6D scores. The same results were seen when the multivariate model was run backwards (data not shown).

**Table 3:** Independent associations with the health-related quality of life measures SF-36, SF-6D and 15D explored in 380 patients with axial spondyloarthritis in outpatient clinics

	SF-36-PCS Range 0–100		SF-36-MCS Range 0–100		15D Range 0–1		SF-6D Range 0–1	
	Adj. B (95% CI)	p	Adj. B (95% CI)	p	Adj. B (95% CI)	p	Adj. B (95% CI)	p
Demographic factors								
Age (years)	−0.01 (−0.08, 0.06)	0.799	0.75 (−0.03, 0.18)	0.161	0.000 (0.000, 0.001)	0.267	0.000 (0.000, 0.001)	0.306
Female	0.19 (−1.54, 1.92)	0.830	0.27 (−2.23, 2.77)	0.834	−0.011 (−0.027, 0.005)	0.192	0.004 (−0.015, 0.023)	0.693
Living alone	0.40 (−1.45, 2.26)	0.669	−0.49 (−3.78, 2.19)	0.718	0.002 (−0.016, 0.019)	0.838	−0.005 (−0.025, 0.016)	0.644
Employment	−1.15 (−3.05, 0.74)	0.233	−2.46 (−5.20, −0.29)	0.079	−0.014 (−0.031, 0.004)	0.135	−0.015 (−0.036, 0.006)	0.164
Education duration								
≤ 10 years	−0.86 (−3.41, −1.69)	0.506	−0.71 (−4.41, −2.97)	0.666	−0.019 (−0.042, 0.005)	0.119	0.018 (−0.010, 0.047)	0.207
11–13 years	−0.01 (−1.68, 1.70)	0.992	0.09 (−2.35, 2.54)	0.701	0.007 (−0.009, 0.023)	0.392	−0.001 (−0.020, 0.017)	0.893
> 13 years	Ref		Ref		Ref		Ref	
Exercise >3 h per week	2.71 (0.21, 4.99)	**0.020**	2.58 (−0.72, 5.89)	0.125	0.036 (0.015, 0.058)	**0.001**	0.053 (0.028, 0.078)	**<0.001**
Exercise 1–3 h per week	2.73 (0.40, 5.06)	**0.022**	1.84 (−1.54, 5.21)	0.284	0.032 (0.010, 0.53)	**0.004**	0.045 (0.019, 0.070)	**0.001**
Exercise <1 h per week	Ref		Ref		Ref		Ref	
Current smoker	−1.07 (−2.81, 0.67)	0.227	−1.40 (−1.12, 3.91)	0.275	0.009 (−0.007. 0.025)	0.284	−0.002 (−0.021, 0.017)	0.842
Alcohol (per week)								
Never	−0.70 (−3.82, −2.23)	0.607	2.74 (−1.62, 7.02)	0.217	0.004 (−0.025, 0.032)	0.798	0.010 (−0.023, 0.043)	0.556
1–6 glasses	−0.85 (−3.49, −1.80)	0.530	4.63 (0.80, 8.46)	**0.018**	0.010 (−0.015, 0.034)	0.428	0.019 (−0.010. 0.048)	0.198
7 or more glasses	Ref.		Ref.		Ref.		Ref.	
BMI (kg/m^2^)	0.23 (−0.16, 0.20)	0.799	0.03 (−0.23, −0.28)	0.827	−0.001 (−0.002, 0.001)	0.447	0.001 (−0.002, 0.002)	0.884
Disease activity								
Measures								
BASDAI (range 0–10)	−0.21 (−0.81, 0.57)	0.729	−0.38 (−1.37, 0.62)	0.459	−0.004 (−0.011, 0.002)	0.201	0.002 (−0.006, 0.010)	0.659
MASES (range 0–13)	−0.09 (−0.32, 0.13)	0.421	−0.02 (−0.35, 0.31)	0.911	−0.001 (−0.003, 0.001)	0.251	0.001 (−0.003, 0.002)	0.885
Health status								
BASFI (range 0–10)	−1.05 (−1.86, −0.25)	**0.010**	1.02 (−0.15, 2.18)	0.086	−0.004 (−0.011, 0.004)	0.348	0.001 (−0.008, 0.010)	0.866
BAS-G (range 0–10)	−0.91 (−1.45, −0.37)	**0.001**	−1.73 (−2.51, −0.95)	**<0.001**	−0.010 (−0.015, −0.005)	**<0.001**	−0.020 (−0.026, −0.014)	**<0.001**
HAQ (range 0–3)	−4.61 (−7.30, −1.91)	**0.001**	−2.00 (−5.91, 1.90)	0.313	−0.051 (−0.076, −0.026)	**<0.001**	−0.054 (−0.084, −0.024)	**0.001**
Damage score								
BASMI (range 0–10)	−0.11 (−0.56, 0.34)	0.629	0.05 (−0.60, 0.69)	0.883	0.001 (−0.003, 0.005)	0.730	−0.002 (−0.007, 0.003)	0.488
Co-morbidity								
Mean total score for co-morbidity (range 0–10)	−0.19 (−1.20, 0.82)	0.715	−0.64 (−2.10, 0.82)	0.388	−0.014 (−0.023, −0.005)	**0.004**	−0.002 (−0.014,0.009)	0.661
Centre								
SSHF (N/Y)	−1.58 (−3.34, 0.18)	0.079	1.47 (−1.08, 4.02)	0.256	−0.005 (−0.021, −0.012)	0.582	−0.013 (−0.032, 0.007)	0.199
R^2^	57.1%		21.5%		60.2%		56.9%	

Adjusted analyses were performed using multiple regression analyses and applying a general linear model using SPSS. Bold *p* values indicate significant differences

Key: *Adj* adjusted unstandardized regression coefficients with 95% confidence interval (CI) and *p*-values; SF-36-PCS, physical component summary scale; SF-36-MCS, mental component summary scale, *BMI* body mass index, *BASDAI* Bath Ankylosing Spondylitis Disease Activity Index, *MASES* Maastricht Ankylosing Spondylitis Enthesitis Score, *BASFI* Bath Ankylosing Spondylitis Functional Index, *BAS-G* Bath Ankylosing Spondylitis Patient Global Score, *HAQ* Health Assessment Questionnaire, *BASMI* Bath Ankylosing Spondylitis Metric Index, *Ref* reference values for statistical comparison

## Discussion

When exploring the associations between demographic- and disease-related variables and HRQoL in patients with ax-SpA, our main finding was that low levels of exercise, impaired physical function (HAQ scores) and impaired general disease-associated well-being (BAS-G scores) were consistently and independently associated with decreased HRQoL across different relevant measures. Interestingly, in the adjusted analysis, apart from exercise intensity, no significant associations were found between demographic variables and the HRQoL measures SF-36-PCS, SF-36-MCS, 15D and SF-6D.

Our patients with ax-SpA reported lower HRQoL (SF-36 scores) compared with norm-based SF-36 reference scores in Norway [35], with the effect size ranging from −0.20 (mental health) to −0.98 (general health). The effect sizes indicate a substantial burden on HRQoL in the patient group, especially within the physical SF-36 domains. Our patients’ SF-36 scores were similar to the scores of Alkan et al. (2013) [15] who assessed HRQoL in Turkish patients with ax-SpA, highlighting the burden of this disease for such patients.

Minor statistically significant differences between female and male patients were only found for some demographic factors (BMI and alcohol consumption), some disease-related variables (swollen joints, entheses score, physical function and skeletal damage score) and some HRQoL domains (SF-36 bodily pain and physical role limitations; Tables 1 and 2). This suggests that the overall burden and the perception of ax-SpA are similar rather than different between genders. In previous studies, women with ax-SpA reported lower HRQoL values than men [13, 14], as well as more pain and more physical consequences of pain [13, 14]. Our data are consistent with those reports as our female patients with ax-SpA reported more bodily pain and reported reduced physical role limitations on the HRQoL measure SF-36.

In patients with ax-SpA, pharmacological anti-inflammatory treatment with NSAIDs and anti-tumour necrosis factor (TNF)-α treatment have been shown to improve physical function and quality of life [38, 39]. The percentage of ax-SpA patients in our study on NSAIDS was 44%, and users of biological DMARDs comprised 23%. This may explain the rather low level of disease activity seen in several measures reflecting this (e.g., BASDAI scores) in our study.

Exercise intensity was identified as being significantly associated with HRQoL across the different measures. Along with targeted treatment, exercise and rehabilitation therapy are cornerstones in the management of ax-SpA, including patients with AS [38–41]. Systematic workouts such as water exercises and exercises for increasing flexibility, muscle strength and cardio-respiratory fitness in ordinary clinical care have been shown to be beneficial [17, 40]. Good physical function implies coping in terms of daily activities and living, which further implies that the patients with ax-SpA can live nearly ordinary lives despite their chronic illness. Exercise is an important part of the holistic approach in treatment and care of this patient type [17, 40].

Co-morbidity was only associated with the 15D measure and not with the other HRQoL measures. Because the items included in this questionnaire are more concrete than those included in the other questionnaires, they may capture the disease-related limitations caused by ax-SpA more specifically [20–23]. Furthermore, the items addressed in the 15D questionnaires might be considered easier to respond to [20, 22], which is also our clinical experience [28]. Another  explanation for the lack of association between co-morbidity and HRQoL might be the low number of co-morbidities in our patients, which seemed to be lower than in previous studies [24].

Back pain is a characteristic symptom in patients with ax-SpA [3]. Pain was only measured by the SF-36 as bodily pain along with physical function measures that might be affected. When we compared the SF-36 bodily pain score with norm-based scores, the effect size revealed a substantial difference between the two groups [35]. These comparisons underline the pain problem in patients with ax-SpA, although it is not site specific. Other HRQoL studies in patients with ax-SpA have also included a visual analogue scale (VAS) [15, 42], and a more specific pain measure might have revealed more nuanced and other findings. On the other hand, the consequence of pain in terms of reduced functioning was extensively covered by the other patient-reported measures in our study.

To our knowledge, this is the first study measuring HRQoL using the utility measures 15D and SF-6D in patients with ax-SpA. Interestingly, in the multivariate models shown in Table 3, the two utility measures—easier to use than the more comprehensive SF-36—performed as well as the SF-36-PCS expressed as the variance (R^2^ **)** and compared better with the SF-36-MCS outcome. This might be interpreted as a test of concurrent validity for the two utility measures [43]. Furthermore, the relatively high variances in HRQoL (except for SF-36-MCS) explained by the demographic- and disease-related variables included in the multiple/adjusted comparisons might also indicate that the independent variables chosen for the analyses (those that are mostly collected in ordinary clinical care) were suitable [37, 43]. On the other hand, there is a question as to whether there were too many independent/explanation variables in the multiple analyses or whether there were overlaps between the questions included in the measures in the independent and dependent variables in the multiple regression model, such as the HAQ and SF-36-PCS measures [22, 23, 44]. The high validity of the utility measures, compared with SF-36 as the gold standard for the assessment of HRQoL, could be of importance, as only feasible HRQoL measures might have the potential to be used as part of the clinical standard in routine clinical care.

The disease-related independent variables that remained as significant associations with HRQoL in the adjusted analyses were all self-reported health or symptom measures. In their conceptual model, Wilson and Cleary linked clinical variables with HRQoL [45]. Their model shows how measures can be thought of as existing on a continuum of increasing biological (e.g., objective ax-SpA disease measures), social and psychological complexity. In this model, HRQoL is influenced by self-reported symptoms of functional status, along with characteristics of the individual and the environment, and not biological and other objective health and disease measures [45]. This seems to have been the case in our study as well.

### Methodological considerations

The strengths of this study were that all the participants were recruited consecutively from two geographically restricted areas and the numbers were sufficient for robust analyses. Further, apart from age, there were few exclusion criteria and there were no differences between the patients included in the study and those who did not want to participate. At the time the data were collected, patients at MHH used significantly more biological DMARDS than did those at SSHF, which might explain some of the differences in self-reported health status. However, in the adjusted analyses, the centres did not turn out to be significantly associated with dependent variables. Therefore, pooling of the patients from the two areas was justified, as there were only small differences between them. Another strength of our study is that we used several validated patient-reported questionnaires and outcomes to cover the patients’ perspectives [20–22, 27].

A major limitation of our study was the cross-sectional study design, which does not permit any causal interpretations. In this regard, our results should be interpreted with caution as we only found associations between dependent and independent variables. Interestingly, when the adjusted models were run backwards, the results remained the same, indicating that our findings are likely robust.

## Conclusions

In patients with ax-SpA, low physical activity (exercising <1 h per week), impaired physical function (higher HAQ scores) and impaired general ax-SpA disease well-being outcomes (higher BAS-G scores) were independently and consistently associated with decreased HRQoL across several measures. This indicates that the two utility measures (15D and SF-6D) reflect and capture HRQoL in patients with ax-SpA to the same extent as the SF-36. Our study has confirmed that ax-SpA negatively influences HRQoL and suggests that physical exercise is important as a specific treatment strategy to maintain and improve HRQoL in patients with ax-SpA.

## Abbreviations

AS: Ankylosing spondylitis
ax-SpA: axial spondyloarthritis
BASDAI: Bath Ankylosing Spondylitis Activity Index
BASFI: Bath Ankylosing Spondylitis Functional Index
BAS-G: Bath Ankylosing Spondylitis Patient Global Score
BMI: Body mass index
CRP: C-reactive protein
DMARDs: Disease-modifying anti-rheumatic drugs
GLM: General linear model
HAQ: Health Assessment Questionnaire
HRQoL: Health-related quality of life
MASES: Maastricht Ankylosing Spondylitis Enthesitis Score
MCS: Mental component summary
MHH: Martina Hansens Hospital
NSAIDs: Non-steroidal anti-inflammatory drugs
PCS: Physical component summary
QALYs: Quality-adjusted life years
REK: Norwegian Regional Committee for Medical Research Ethics
SSHF: Sørlandet Hospital
